# Envoy

**Published:** 1913-07

**Authors:** Charlotte Becker


### Envoy.

Say not, because he did no wondrous deed,
Amassed no worldly gain,
Wrote no great book, revealed no hidden truth,
Perchance he lived in vain.

For there was grief within a thousand hearts
The hour he ceased to live;
He held the love of women, and of men—
Life has no more to give.

—*Charlotte Becker.*

